# Measurement of saccadic eye movements by electrooculography for simultaneous EEG recording

**DOI:** 10.3758/s13428-019-01280-8

**Published:** 2019-07-16

**Authors:** Yingxin Jia, Christopher W. Tyler

**Affiliations:** 1 Brain Imaging Center, Smith-Kettlewell Eye Research Institute, San Francisco, CA USA; 2 Division of Optometry and Visual Sciences, City, University of London, London, UK

**Keywords:** Saccades, Eye movements, EOG, Free-viewing, Blink detection

## Abstract

Eye movements are an important index of the neural functions of visual information processing, decision making, visuomotor coordination, sports performance, and so forth. However, the available optical tracking methods are impractical in many situations, such as the wearing of eyeglasses or the presence of ophthalmic disorders, and this can be overcome by accurate recording of eye movements by electrooculography (EOG). In this study we recorded eye movements by EOG simultaneously with high-density electroencephalogram (EEG) recording using a 128-channel EGI electrode net at a 500-Hz sampling rate, including appropriate facial electrodes. The participants made eye movements over a calibration target consisting of a 5×5 grid of stimulus targets. The results showed that the EOG methodology allowed accurate analysis of the amplitude and direction of the fixation locations and saccadic dynamics with a temporal resolution of 500 Hz, under both cued and uncued analysis regimes. Blink responses could be identified separately and were shown to have a more complex source derivation than has previously been recognized. The results also showed that the EOG signals recorded through the EEG net can achieve results as accurate as typical optical eye-tracking devices, and also allow for simultaneous assessment of neural activity during all types of eye movements. Moreover, the EOG method effectively avoids the technical difficulties related to eye-tracker positioning and the synchronization between EEG and eye movements. We showed that simultaneous EOG/EEG recording is a convenient means of measuring eye movements, with an accuracy comparable to that of many specialized eye-tracking systems.

A wide variety of human brain functions have been studied with event-related analyses, in the form of averaged event-related potentials (ERPs). In the vast majority of those experiments, visual processing has been examined with large eye movements precluded, by asking participants to hold their gaze still in order to maintain fixation, and to refrain from blinking while the stimuli are flashed near the fovea. To further prohibit saccadic eye movements, visual stimuli are often presented for a short duration within the saccadic reaction time or at a small stimulus size.

However, the practice of precluding oculomotor activity in most electroencephalographic (EEG) studies eliminates a major component of visual information processing. It has long been established that the eyes produce a continual stream of saccadic eye movements to bring the fovea to bear on aspects of interest in the visual scene. In addition, confining experiments to conditions without eye movements cannot elucidate the cortical mechanisms involved in control of the eye movements themselves.

To investigate the brain activity specific to eye movement control, we need to measure brain responses through EEG signals and eye movements simultaneously. It has been justified that combining EEG and eye tracking is possible (Nikolaev, Meghanathan, & van Leeuwen, 2016). Currently, there are many different methods to measure eye movements, with varying difficulties and costs. One option is infrared video-based eye tracking systems, which can achieve good accuracy. But concurrent eye tracking in the process of measuring EEG signals requires the management of technical issue related to synchronization. Special trigger pulses (Baccino & Manunta, 2005) need to be sent to both the EEG and eye-tracking systems to achieve synchronization. Such eye trackers can be difficult to set up for certain individuals, particularly those needing to wear spectacles or low-vision aids for optimal vision.

Another option to measure saccadic eye movements is electric potential measurement by means of electrooculography (EOG). This reliable technique for measuring saccadic eye movements is based on the mechanism that the eyes are the origin of a steady potential field that can be modeled as a dipole with its positive pole at the cornea and negative pole at the retina. Electrodes placed in the periocular region are used to measure these electric potentials. If the eyes move from the center position to a peripheral location, the cornea approaches one electrode while the retina approaches the opposing one. This change in the orientation of the dipole results in a change in the measured EOG signal. The EOG technique has the advantage in concurrent eye tracking that the simultaneous measurements of EEG signals and eye movements by EOG can be achieved by using the same high-density electrode net. This effectively avoids the technical difficulties related to eye-tracker positioning and synchronization between EEG and eye movements.

Some previous algorithms have been developed for the detection of saccades in EOG. Niemenlehto (2009) employed a detection approach of constant false alarm rate to detect saccades during the analysis of EOG signals. Pettersson et al. (2013) used the temporal derivative of the EOG eye movement signals for determining the threshold to detect saccades and blinks. Behrens, MacKeben, and Schröder-Preikschat (2010) utilized the deviation of eye-movement acceleration values from the EOG signals. Toivanen, Pettersson, and Lukander (2015) considered unsupervised training to establish a real-time probabilistic algorithm for detecting saccades. However, none of these studies systematically measured the saccades beyond the central 10° field using seven eye movement EOG electrodes, or assessed the linearity of the saccade trajectories, or estimated the bivariate error function of their measurements of the resulting saccade endpoints in *x*, *y* space, or compared them with the errors reported for other techniques.

This present study was designed to assess the efficacy EOG signals measured from the high-density EEG electrode net for the accurate analysis of the amplitudes and directions of saccades. This article also provides a sophisticated analysis of the nature of blink responses through detailed measures of EOG signals around eyes.

## Method

### Participants

We recruited seven participants for the simultaneous measurement of EEG and EOG (five male, two female; age range: 27–37 years), all with normal or corrected-to-normal vision. They were each informed about the purpose of the study and the procedures, and had provided a written consent before the measurements. All procedures conformed to the Declaration of Helsinki and were approved by the Smith-Kettlewell Institutional Review Board.

### Stimulus presentation

The stimuli were presented on a 19-in. color monitor (Sony LCD Color Monitor, MODEL: SDM-S93, Sony Electronics, Inc. FL, refresh rate 100 Hz, resolution 1,280 × 1,024 pixels) at a viewing distance of 33 cm. A 5×5 stimulus array was configured in a square region of 34° × 34°, subtended in the center of a dark screen with a background luminance of 3.06 cd/m^2^. A red target (size ~ 0.5°) appeared randomly within this square grid at 25 possible stimulus locations.

### Experimental procedures

**Fig. 1:**
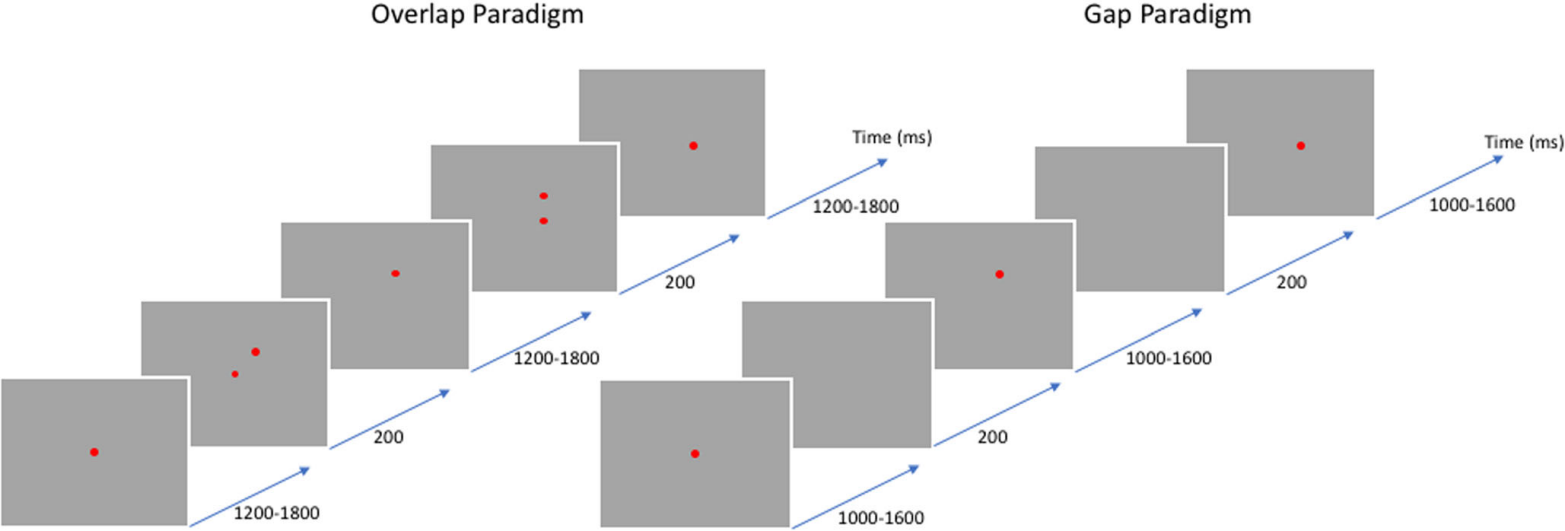
The experimental paradigms (see the text for details)

The participants performed the fixation task illustrated in Fig. 1. The experimental blocks always started with the red target in the center of the screen, with each red dot target appearing at one of the calibration locations 1,200–1,800 ms after the appearance of the previous one. The stimulus paradigms followed the classic work showing that saccade latency is affected by the timing of the trigger events initiating the saccadic eye movements (Saslow, 1967). In one condition (*saccadic overlap paradigm*), the preceding target stayed on for another 200 ms after the next target had appeared. In the other condition (*saccadic gap paradigm*), instead of both staying on, the first target was removed 200 ms before the appearance of the second target. The saccade latency is typically about 100 ms longer for the overlap than for the gap paradigm. The experimental participants were required to saccade to each target location and to maintain fixation until the next target appeared. A chin-rest was used to stabilize the head throughout the experiments. In both conditions, the choice of sequential locations was randomized across the array, subject to the constraint that the length of each saccade in terms of combined vector was limited to no more than 24° (to avoid nonlinearities of the trajectory reconstruction beyond that range). Thus, the largest saccade was equal to the length from the center to a corner of the stimulus array. Each experimental block consisted of 96 saccade trials. The participants were allowed a 5-min break after each block presentation, to relieve any peri-ocular muscle fatigue.

### EEG recordings

EEG data were recorded using an EGI Geodesic Netstation high-density, whole-head recording system (Electrical Geodesics, Inc., Eugene, OR), which incorporates 128 electrodes distributed around the head and face, in addition to the scalp electrodes. The impedance of all electrodes was maintained below 60 kΩ according to the recommended value for this system. The data were recorded at a sampling rate of 500 Hz and band-pass filtered between 0.01 and 250 Hz. The recorded signals for all electrodes were referenced to the vertex electrode (Cz).

### Eye tracking

Eye movements were recorded through the measurement of electric potentials (EOG) using the Netstation electrodes located around the eyes, which included channels appropriate for recording vertical and horizontal EOGs. The vertical EOG for each eye was derived from the differential signal between electrodes located in the infraorbital ridge of the eye and the one immediately above the eye (#126 and #8 for the right eye, and #127 and #25 for the left eye in the Netstation coding), and the horizontal EOG was derived from the differential signal between electrodes located in the middle of both eyes (#17) and the one near the outer canthus of each eye (#1 for the right eye and #32 for the left eye).

### Preprocessing and analysis

All recorded EEG data were preprocessed and analyzed in Matlab (Mathworks, Natick, MA). Electrical mains 60 Hz and monitor refresh 100-Hz pickup were first removed by an innovative frequency splatter filtering procedure (Tyler & Likova, 2017). Then, EEG data were low-pass filtered at 100 Hz and re-referenced to the average activity of the 126 scalp electrodes. The signals from two electrodes near each ear (#113 near the right ear, and #49 near the left ear) were eliminated due to a high noise level. Two kinds of analyses were performed. One used the stimuli as the time markers, and the other analyzed the eye-movement waveforms as if they were from free-viewing condition, without reference to the time markers. For the saccade detection with a target cue, the recordings were segmented into 1.5 s epochs from – 500 to + 1,000 ms around the onset of each target. The data in each epoch were then high-pass filtered at 0.2 Hz to remove low frequency drift, and the horizontal and vertical EOG signals in each epoch were each baseline-corrected to the level of the stimulus interval of 100 ms just preceding the stimulus. The vector sum of the baseline-corrected horizontal and vertical EOG signal amplitude was used to detect saccades.

### Cued saccade detection

The custom software for saccade detection with a target cue operates as follows. A recording of combined EOG signals of horizontal and vertical EOG were processed continually throughout each epoch. The onset of saccades was specified analytically as any point of significant deviation from the local noise level, as defined by the running average of the standard deviation (RASD) of the combined EOG signal amplitude over the previous 500 ms (Behrens et al., 2010). This principle was implemented as the point at which combined EOG signal amplitude first exceeded a criterion level of 5 × RASD. Specifically, the RASD was obtained by segmenting each 500-ms period into 50-ms-long overlapping data epochs, advancing in 25-ms steps from – 500 to 0 ms, where 0 is the onset of a target. Thus, there were 19 overlapping windows with 19 standard deviations during this 500-ms pretarget period. The RASD was then computed as the average of the mean of the lowest ten standard deviations (*SD*s) over all the artifact-free epochs. To define the saccade onset, the criterion time point for the detected saccade was backdated to a time point of 25 samples (50 ms) before the currently inspected point. Once the onset of the saccade was found, the EOG data were resegmented into the range from – 400 to 400 ms relative to the saccade onset for the detection of saccade offsets, as follows: The offsets of saccades were obtained through the velocity function of the normalized vector sum of the horizontal and vertical EOG signal amplitudes from – 400 to 400 ms relative to the onset of saccade. The saccade offset was then defined as the first point at which the value of the velocity function fell to less than 15% of its maximum velocity.

### Uncued saccade detection

Saccades could also be detected without applying the target cues, as a test of the capability of detecting uncued saccades, corresponding to free-viewing conditions. To assess the ability to identify the saccades without cueing, this uncued saccade detection procedure was based on processed the continuous horizontal and vertical EOG recorded for the entire experimental block (~ 2.5 min), without respect to the target presentation. First, the whole block was divided into intervals of 100 samples (200 ms). The presence of a saccade was assumed to generate an increase in the *SD*. All pairs of adjacent artifact-free EOG intervals, the second with a relatively higher *SD* than the first, were then identified throughout the recordings for the saccade detection analysis. For all samples in the second interval of the pair, the onset of saccades was then defined analytically as any sample point whose value deviated a criterion level of 5 × *SD* from the average *SD* in the 200-ms period immediately preceding that sample. The whole horizontal and vertical EOG sequences were probed separately in this way until all the specified saccades were detected, regardless of direction. This algorithm is applicable under any free-viewing conditions.

The effectiveness of the uncued procedure was assessed in terms of its sensitivity and specificity. *Sensitivity* was specified as the proportion of saccades detected with this uncued procedure in an experimental run that actually contained cued saccades to provide the ground truth for the saccade occurrence. *Specificity* was defined as the proportion of correct rejections provided by the same procedure applied to intertarget intervals that did not contain saccade (i.e., from at least 500 ms after the preceding target to just before the next target).

### Calibration procedure

The horizontal and vertical saccade amplitudes were calibrated with a three-parameter affine scaling function of both the horizontal and vertical EOG signals, based on the target locations. The saccade amplitudes were derived from the EOG signals for the differences in voltage between the onsets and offsets of the saccades (obtained as above). There were separate sets of slope and intercept scaling factors for the horizontal and vertical affine scaling functions. The three parameters for each of horizontal and vertical EOG scaling functions are dominant slope, nondominant slope and intercept. Therefore, both the horizontal and vertical EOG signals can be represented as follows:

1 $$ EOG=a\ast \varDelta X+b\ast \nabla Y+c $$

where *a* and *b* are the parameters representing two slopes, and *c* is a parameter representing the intercept. ∆*X* and ∆*Y* are the differential target displacements along the *x*- and the *y*-axis, respectively. Whether *a* or *b* is the dominant slope depends on the direction being calibrated. When calibrating horizontal EOG amplitude, *a* is the dominant slope, whereas *b* is the dominant slope for vertical EOG calibration. Only the dominant slope and intercept were maintained as scaling factors, such that horizontal EOG signals can be expressed on the basis of the target locations, as follows:

2 $$ hEOG={a}_h\ast \varDelta X+{c}_h $$

where *a*_*h*_ and *c*_*h*_ are a set of slope and intercept scaling factors for this horizontal affine scaling function. The vertical EOG signals can be expressed as follows:

3 $$ vEOG={b}_v\ast \varDelta Y+{c}_v $$

where *b*_*v*_ and *c*_*v*_ is a set of slope and intercept scaling factors for this vertical affine scaling function. With regard to the stability of the calibration over time, these affine calibration expressions were assumed to be specific to the electrode locations. Therefore, in common with typical eye movement calibration procedures, the affine transformation was invariant over time once established for a given set of electrode placements.

### Principal component analysis (PCA)

To identify eye movement control components from the data, we performed an iterative form of PCA on the matrix of saccade-defined epochs to avoid the orthogonality constraint, which is unlikely to represent the physiological organization of the EEG responses. PCA in general is a blind source separation algorithm that enables the separation of statistically distinguishable sources from multidimensional data, and was implemented using the singular value decomposition (svd) function in MATLAB. In its full implementation, svd derives the set of *n* orthogonal vectors characterizing the input EEG data matrix, where *n* is the number of parallel signal vector inputs (matrix rows). To distinguish the directions of the saccades for the PCA analyses, all the saccades were separated into four direction sectors according to their closest adjacent cardinal direction (left, up, right and down) before applying the PCA. For each participant individually, the EEG amplitudes were averaged in each sector, yielding four 2-D time-by-electrode matrices. To get each principal component, the four two-dimensional (2-D) time-by-electrode matrices for the four conditions were concatenated in the second (electrode) dimension to get an omnibus 2-D matrix. A full svd on this omnibus 2-D matrix yielded orthogonal temporal component waveforms and spatial distributions. Only the primary temporal component (eigenvector) and spatial distribution were retained from the first svd of the concatenated the data, the rest of the temporal components obtained being discarded. This first eigenvector, weighted by its eigenvalue, was then subtracted from all the data and a second svd was implemented on the remaining data to yield further temporal PCA component waveforms and spatial distributions. Again, only the first primary temporal component and spatial distribution were retained to form the second iterative principal component. A series of sequential svd was performed, each on a remaining data, until eight iterative components were reached. This whole process was called *iterative PCA (iPCA)*. It had the advantage over the classic single PCA that the constraint of orthogonality among the produced principal components by a single PCA was eliminated by the series of iterative PCA, thus providing the capability of revealing more physiological components of the eye movements and cortical process. In addition, multiple secondary peaks in an extracted component waveform might be eliminated through the iterations of PCA, which is easier for component interpretation.

## Results

### Blink artifact

**Fig. 2:**
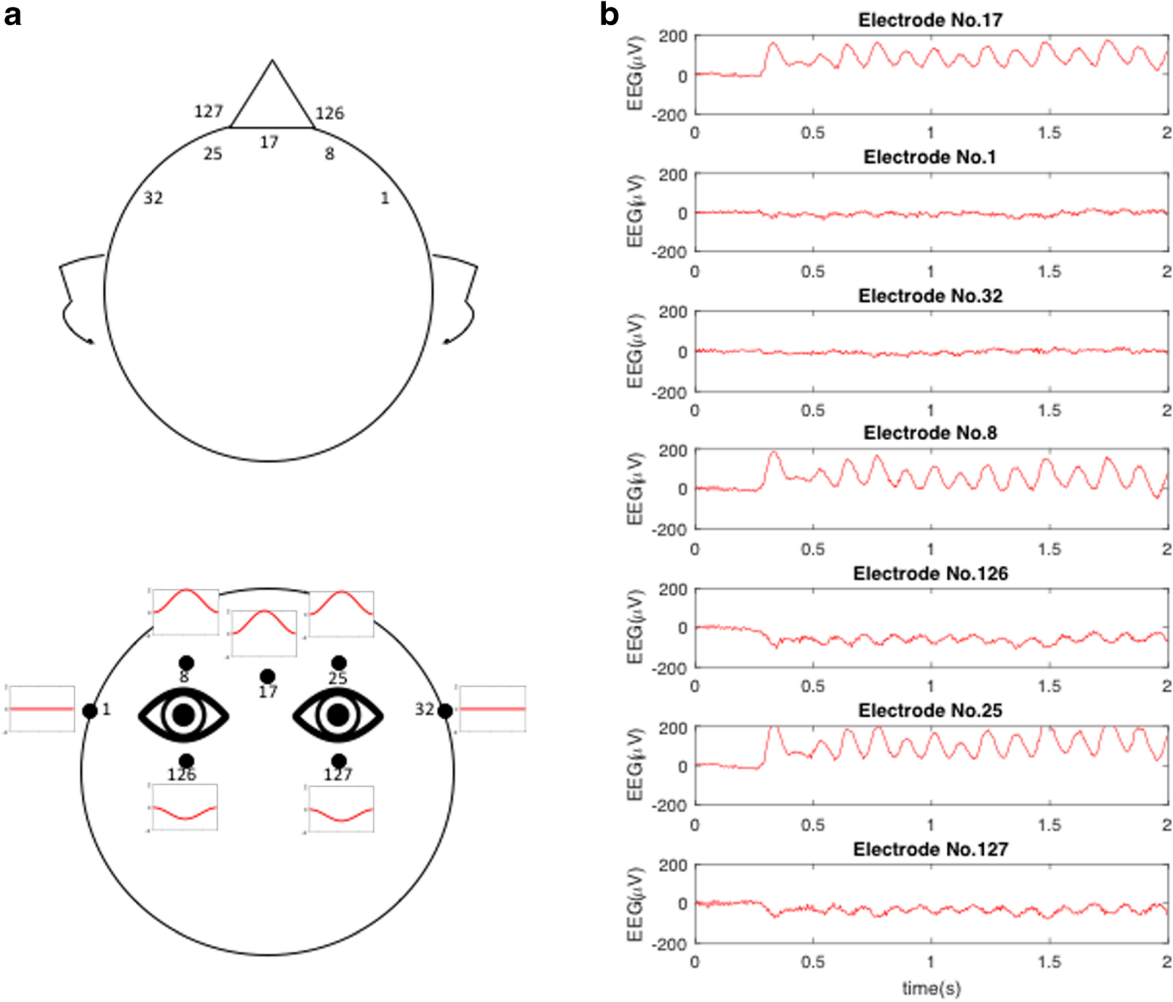
(A) Illustration of the positions of the face electrodes, with numbering from the 128-electrode EGI net. (B) Blink potentials from one participant who was required to blink as fast as possible, resulting in approximately 8-Hz oscillations

During the EEG recording, large deflections were easily seen at the vertical EOG electrode sites (#126, #8, #127, and #25 in the Netstation coding) whenever blinks occurred. Therefore, to determine the nature of the blink artifacts, we added a short EEG recording with one participant to examine the blink potentials. During a 2-s EEG recording, the participant was asked to blink as fast as possible, which provided a frequency of ~ 8 Hz. Figure 2A illustrates the exact locations of seven EOG electrode sites (#17, #1, #32, #8, #126, #25, and #127 in the Netstation coding), in both top and frontal views. The average forms of the measured blink potentials are diagrammed in Fig. 2B. During blinks, this shows that there were large (~100-*μ*V) *positive* deflections above the eyes (#17, #8, and #25), *negative* deflections with a smaller amplitude (~20 *μ*V) below the eyes (#126 and #127), and almost *zero* deflections at the temples (#1 and #32). The question of what electrical source would give rise to this pattern of signal changes during the blink action of closing the eyelids to cover the eyeballs is addressed in the Discussion.

### The origin of the EOG signal

**Fig. 3:**
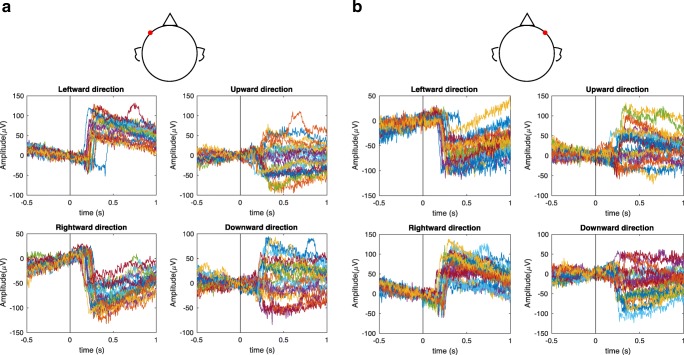
Event-related potentials during left, right, upward, and downward saccades with different amplitudes, as measured at (A) the left temple electrode and (B) the right temple electrode for one participant during the overlap paradigm. The responses are aligned to the onset of the stimulus at time 0

The electrical activity changes generated by saccadic eye movements were recorded from electrodes in the periocular regions. Due to the standing corneo-retinal dipole charge of the eyeball, a change in the orientation of the eyeball generated corresponding electrical changes at the electrodes. Figure 3 shows the electrical potentials recorded during leftward, upward, rightward, and downward saccades measured at each lateral temple electrode for the overlap paradigm. The electrical potentials for each cardinal direction measured at the right temple electrode (#1 in the Netstation coding) are shown in Fig. 3B, and the potentials measured at the left temple electrode (#32 in the Netstation coding) are shown in Fig. 3A. Zero in the time axis means the onset of the stimuli. The large eye movements result in conspicuous transient responses in the EOG signal, reflecting the orientation change of the corneo-retinal dipole as the eye moves to different locations in the stimulus array.

**Fig. 4:**
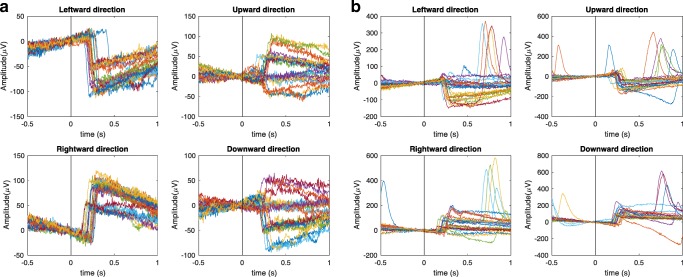
The horizontal and vertical electrooculography (EOG) signals for one participant during the overlap paradigm are aligned to the onset of the stimulus at time 0. The horizontal EOG (A) is obtained from subtraction between the EOGs recorded from the electrodes placed at the right and left temples, whereas the vertical EOG (B) is obtained from subtraction between the mean EOGs recorded from the electrodes placed above and below both eyes

The differential horizontal and vertical EOG signals, respectively, for the overlap paradigm caused by saccadic eye movements with arbitrary directions are shown in Figs. 4A and 4B. The differential horizontal EOG signals were given by the electrical potentials from the right temple electrode subtracted from the left temple electrode. The differential vertical EOG signals were given by the mean electrical potentials of both eyes obtained from the difference between the electrodes above and below both eyes combined. Note that, in accordance with the findings from Fig. 2, blink artifacts were seen only from the vertical EOG (Fig. 4B)—that is, from the electrodes above and below the eyes. Also note that this participant exhibits the common trait of frequent blinking following the execution of a saccade but rarely during a saccade. The analysis of the saccade epochs could therefore proceed without significant interference from blink artifacts.

### Detection of saccades using EOG with a target cue

The overall goal of the methodology was to be able to detect saccades occurring in any direction. The first step in defining the occurrence of the recorded saccades with a target cue was to combine the horizontal and vertical saccade traces to provide for detection of the onsets of the eye movements. The combined EOG signals are a vector sum of baseline-corrected horizontal and vertical EOG signals, as follows:

4 $$ EO{G}_{combined}=\sqrt{hEO{G}^2+ vEO{G}^2} $$

**Fig. 5:**
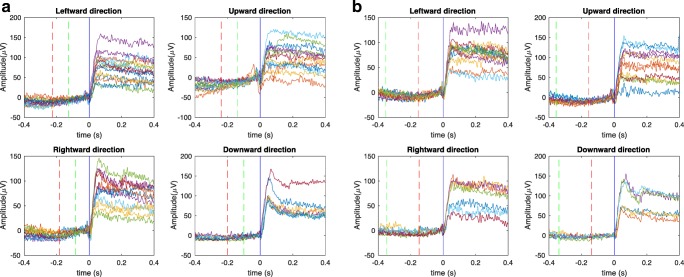
Vector-combined electrooculography (EOG) signals, with the computed saccade onsets aligned at time 0 for (A) the overlap paradigm and (B) the gap paradigm for one participant.

The amplitudes of the combined EOG signals were then used to detect saccades with a custom algorithm (see the Cued Saccade Detection section). The blink-free EOG waveforms after saccade detection are shown in Fig. 5. These EOG waveforms were truncated into epochs from – 400 to 400 ms, with the onsets of saccades at zero. Figure 5A shows the truncated EOG waveforms for the overlap paradigm, and Fig. 5B shows the same for the gap paradigm. The red dashed lines shown in Fig. 5 indicate the average current target onset times, whereas the green dashed lines indicate the average previous target offset times. It can be seen that the average saccadic reaction times, from the onset of the targets (red dashed lines) to the onset of saccades (blue solid lines), are approximately 215.08 ± 6.72 ms and 151.17 ± 5.66 ms for the overlap and gap paradigms, respectively. Thus, the reaction times are relatively longer in the overlap paradigm, implying that the presence of the previous target during a saccade latency delays the saccade (Clark, 1999; Saslow, 1967). This was assessed by a paired *t* test of saccadic reaction times between the overlap and gap paradigms across seven participants, which revealed that the difference in saccadic reaction time between the overlap and gap paradigms was statistically significant (*t* = 6.83, *p* = .00048).

We also noticed that there was a presaccadic blip in the EOG signals (Harrison, Mattingley, & Remington, 2012; Klostermann et al., 1994), which might be due to receptive fields shifting to the next location in the stimulus grid prior to the onset of a saccade.

### Saccade detection under free-viewing conditions

**Fig. 6:**
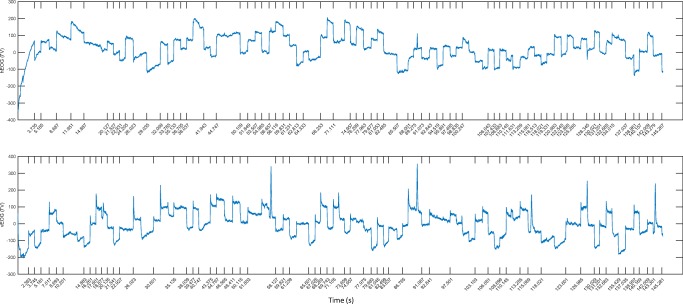
Horizontal (top) and vertical (bottom) electrooculography (EOG) signals, with saccade onset times detected by the free-viewing detection algorithm marked by ticks, for an experimental block in the overlap paradigm

The saccades could also be detected using EOG when there were no target cues. The amplitudes of the horizontal and vertical EOG signals, in microvolts, were used to detect saccades separately with the algorithm described in the Uncued Saccade Detection section. The two EOG waveforms from one participant, with computed saccade onset time ticks, are shown in Fig. 6 for the overlap paradigm. The whole recording for this block lasted for 145.28 s. The sensitivity and specificity of the saccade detection using EOG signals for this recording were 94.79% and 98.96%, respectively, on the basis of 96 saccades.

### Calibration

**Fig. 7:**
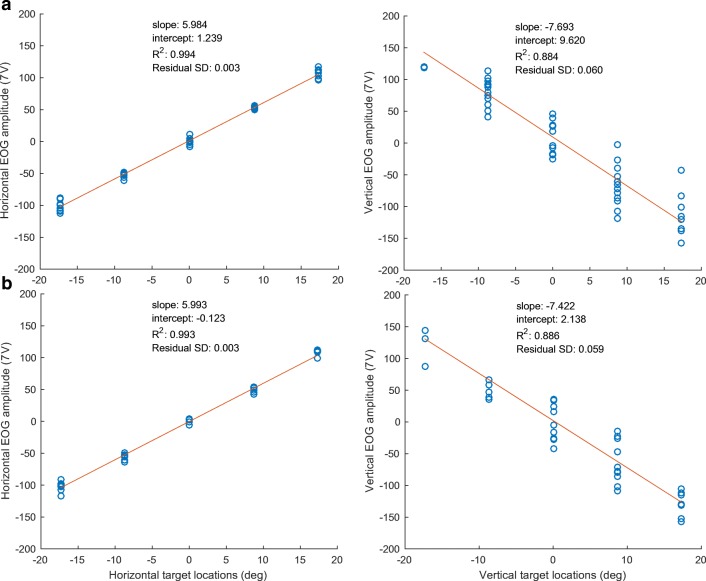
Parameters obtained in the calibration procedure, separately for both the horizontal and vertical dimensions, for (A) the overlap paradigm and (B) the gap paradigm

The key function of saccade recording using EOG is to identify the location of fixation in the target region, requiring accurate calibration of the saccade signals to the target space. To assess the accuracy of the EOG signals, we obtained the mapping from the output of the EOG signals to a set of gaze points, which were the places where the current target was located on the screen. The procedures assessed the degree to which the horizontal and vertical EOG amplitudes were proportional to the horizontal and vertical target locations, respectively. Figures 7A and 7B show how accurate the measured horizontal and vertical EOG amplitudes are in the overlap and gap paradigm, respectively. The saccade amplitudes measured by EOG signal amplitudes were fit with an affine scaling function of the target locations (see the Calibration Procedure section). The horizontal and vertical EOG signal amplitudes were fitted separately in order to obtain a separate set of scaling factors for each direction (parameters *a*, *b*, and *c* in Eq. 1).

As is shown, in the overlap paradigm, the calculated slope and intercept in the horizontal prediction (parameters *a*_*h*_ and *c*_*h*_ in Eq. 2) were 5.984 *μ*V/deg and 1.239 *μ*V, respectively, whereas the slope and the intercept in the vertical prediction (parameters *b*_*v*_ and *c*_*v*_ in Eq. 3) were – 7.693 *μ*V/deg and 9.620 *μ*V, respectively. The coefficients of determination, *R*^2^, for the horizontal and vertical predictions are .994 and .884, respectively, with residual standard deviations of 0.318% and 5.981%.

These fits reveal that, following affine transformation, there is a highly linear relationship between the target locations and the saccade amplitudes predicted from the EOG signals horizontally, and to a lesser degree vertically.

For future projects on saccade detection in free-viewing conditions, the obtained parameters (dominant slopes and intercepts) from the procedure for calibration of the EOG signals to the target locations should be factored. Therefore, the vector combined saccade amplitudes can be expressed as follows:

5 $$ Amplitud{e}_{combined}=\sqrt{{\left(\frac{hEOG-{c}_h}{a_h}\right)}^2+{\left(\frac{vEOG-{c}_v}{b_v}\right)}^2} $$

where *a*_*h*_ and *c*_*h*_ are the dominant slope and intercept of the three-parameter affine scaling function for the horizontal prediction, and *b*_*v*_ and *c*_*v*_ are those for the vertical prediction.

**Fig. 8:**
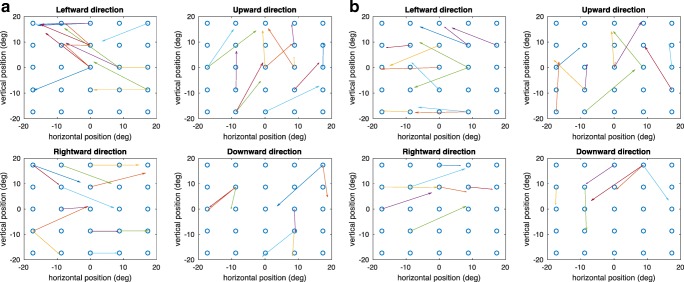
Calibrated saccade trajectories for each cardinal direction for (A) the overlap paradigm and (B) the gap paradigm

**Fig. 9:**
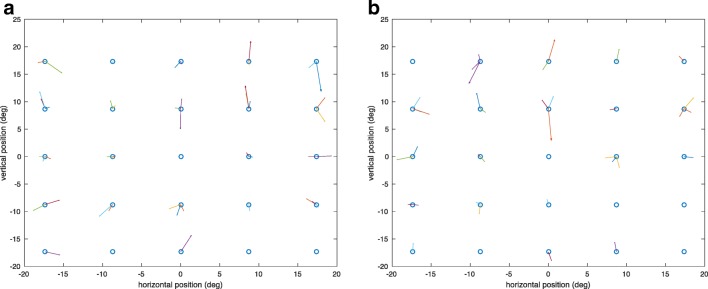
Errors of calibrated saccade endpoints around each target location for (A) the overlap paradigm and (B) the gap paradigm

To visualize the results of the calibration, the randomized saccade trajectories obtained from the calibration are shown in a 5×5 stimulus array. Figures 8A and 8B show the calculated random saccade trajectories expressed in terms of saccade amplitudes for each cardinal direction for the overlap and gap paradigm, respectively. The start point of the saccade trajectory is selected at random. The length of the randomized saccade has the constraint that it is within half of the diagonal of the stimulus array. This helps avoid nonlinearity of the trajectory reconstructions. To illustrate the achievable accuracy of the calibration, the 2-D errors of the saccadic endpoints around each target location are plotted. Each saccade trajectory was displaced so that the start points were aligned at the center of the stimulus grid. The error vectors pointing from the target locations toward the endpoints of the saccades for the overlap and gap paradigm are shown in Figs. 9A and 9B, respectively. In terms of the accuracy of eye position estimation from a given EOG signal, the residual errors across the grid averaged 0.75° and 1.38° in the horizontal and vertical directions, respectively, for the overlap paradigm (Fig. 9A). For the gap paradigm (Fig. 9B), the averaged residual errors across the stimulus grid are 0.81° and 1.60° in the horizontal and vertical directions, respectively.

**Fig. 10:**
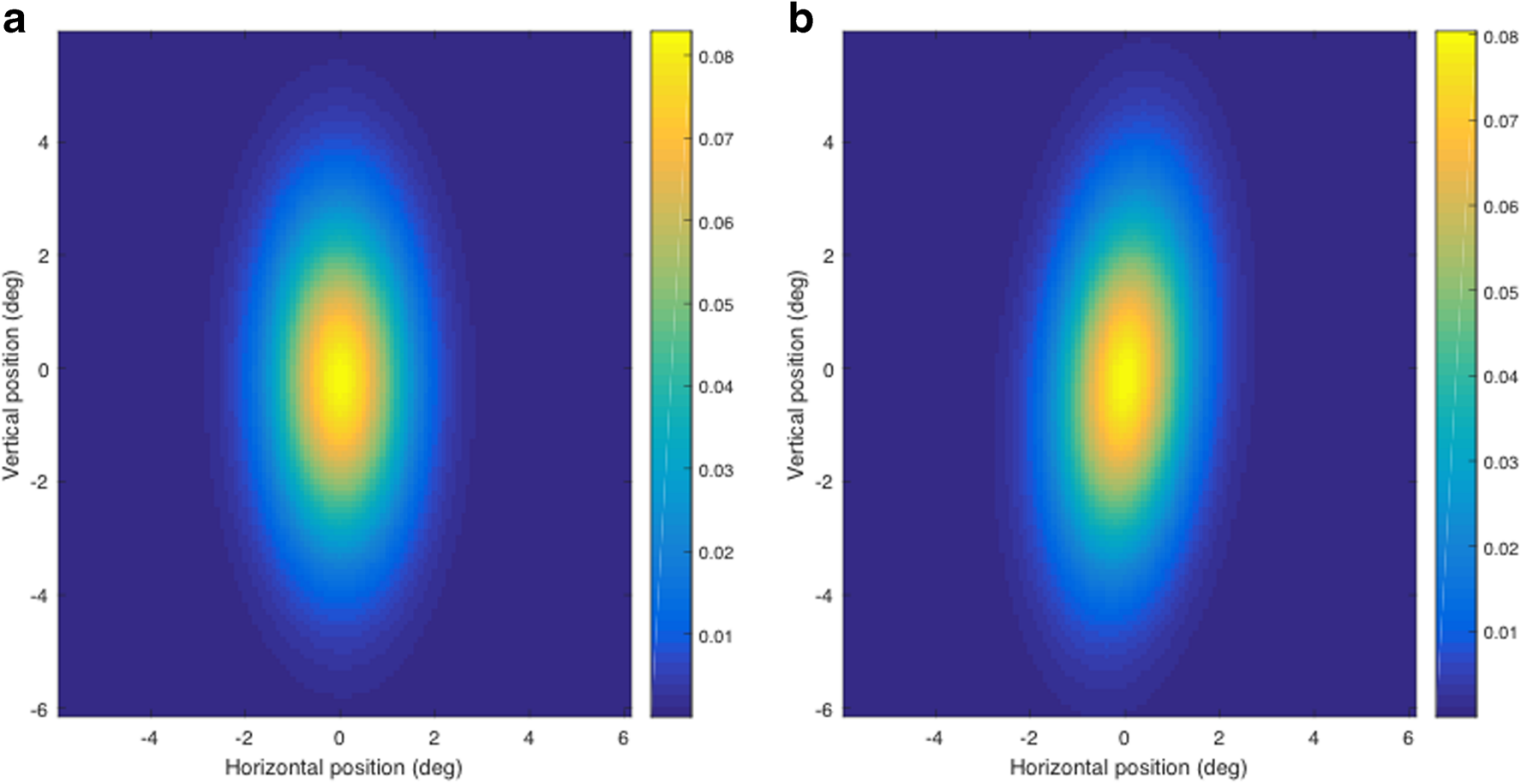
Heat maps of net errors of eye gaze calibration using the electrooculography (EOG) signals for (A) the overlap paradigm and (B) the gap paradigm

The average errors of these calibrated EOG signals across the 34° field of that calibration targets can also be shown as heat maps of the error distributions (Fig. 10), illustrating that the typical error for EOG eye-tracking is comparable to or lower than that of many dedicated eye-tracking systems.

### EEG analysis using PCA

**Fig. 11:**
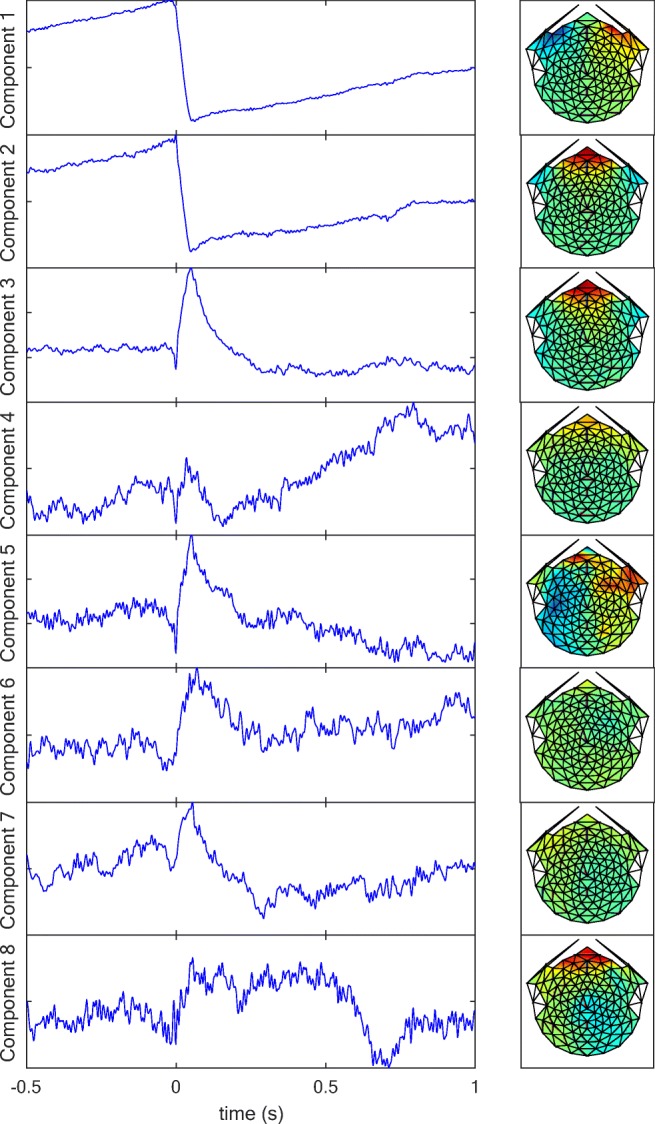
Eight iterative components obtained from an iterative form of principal component analysis

Applying an iterative form of PCA (see the Principal Component Analysis section) on the saccade-defined whole-head EEG data revealed a set of meaningful, dissociable response components in the saccade-triggered potentials, shown in Fig. 11. The PCA was performed on the aligned waveforms, as in Fig. 5. The first two iterative components in Fig. 11 suggest horizontal and vertical saccades with their expected asymmetric and symmetric frontal locations, respectively. (Note that, by the nature of PCA analysis, the inverse pattern in each case for both the temporal and spatial response patterns would be associated with opposite directions of the saccades—left vs. right and upward vs. downward.) Components 3 and 4 indicate a blink response localized in the frontal area, where the fourth component gives a slow component of this response. The localization of Component 5 suggests that it has a lateralized frontal eye field (FEF) origin, with a positive sign on the right and a negative sign on the left, relative to the activation peak. (The inverse property of PCA would again incorporate the inverse pattern for both the temporal and spatial waveforms.) Both Components 6 and 7 show deep brain oculomotor signals, but with different focuses. The last component shown in Fig. 11 reveals a sustained eyelid response.

## Discussion

The novel approach to the accurate recording of eye movements developed in this article is to record EOG simultaneously with scalp EEG recording, to provide an accurate and convenient means of eye tracking without specialized eye-tracking equipment. Two interesting issues are discussed here. One is to compare the accuracy and linearity of EEG/EOG measurement with those of conventional video-based eye-tracking systems. The other is to consider the nature of the blink response measured by the EOG.

### General utility of the EEG/EOG approach to eye tracking

There is a general trend toward combining eye-tracking capability with other types of brain imaging, such as functional magnetic resonance imaging and EEG. The use of dedicated eye trackers in this situation is both cumbersome and unreliable, since they can take a while to set up and do not work for all eye configurations. Moreover, they are particularly challenging for use with those who wear spectacles or low-vision aids. In the case of EEG recording of brain activity, it is greatly advantageous to have a technique for recording eye movements that does not require the use of additional equipment, but is derived directly from the same EEG electrode net as the brain signals. The combined EEG/EOG approach will work for any eye, regardless of color or optical quality, and in combination of the regular refractive devices or optical aids.

### Comparison of EOG and video-based tracking of eye movements

As the calibrated saccadic direction vector results show, the EOG signals recorded simultaneously with EEG by using the high-density electrode net can attain results as accurate as many video-based eye tracking devices. The average angular accuracy reported across an array of detection techniques used for various types of eye tracking was 1.56° ± 0.23° (Hansen & Ji, 2010). In comparison, the present mean residual errors across the current stimulus grid for both the overlap and gap paradigms measured by EOG signals are 0.78° ± 0.03° and 1.49° ± 0.11° in the horizontal and vertical directions, respectively. These error ranges are thus in the lower end of those of typical eye-tracking technology.

The better performance for the horizontal EOG measurements in comparison with vertical EOG signals confirms a previous EOG study (Acuña, Aqueveque, & Pino, 2014). The recorded EOG potentials calibrated into gaze angle in degrees was more accurate for the horizontal direction than for the vertical direction. The calibration results, shown in Fig. 8, also indicated more dispersion in the vertical EOG signals, suggesting more variability than the corresponding horizontal EOG signals. One factor that might account for this greater variability is movement of the eyelid relative to the eyeball during saccades, which, as we showed in the first section, can generate substantial potentials independent of the eyeball rotation per se that could add noise to the vertical (but not the horizontal) EOG signal. In addition, there may be an effect of the more active muscles located in the eyebrows and the cheeks where the vertical EOG electrodes were placed, affecting the vertical EOG signals.

The data also show that both horizontal and vertical EOG signals provide excellent linearity up to at least 17°, since the extension in the stimulus grid reached to 17° in each of four cardinal directions. This is consistent with Acuña et al. (2014), who reported linearity up to 50°, which is a better linearity than video-based eye-tracking systems, with linearity only up to 25° (Bahill, Clark, & Stark, 1975).

As we mentioned in the introduction, the EOG technique used in this study has particular advantages as compared with video-based methods in the measurement of eye movements. The EOG system can be used with any kind of eyewear, including regular spectacles and low-vision aids. In addition, it does not require a fixed head position, allowing the investigation of natural gaze behavior, to the extent that the EOG recording system allows free head movements and is portable.

### Blink response

Although some previous studies have shown that blink artifacts are caused by the cornea being *insulated* from the peri-ocular regions (Faes, van der Meij, de Munck, & Heethaar, 1999; Fish & Geddes, 2009), others have suggested that the eyelids act as a *conductor* during blinks (Antervo, Hari, Katila, Ryhänen, & Seppänen, 1985; Matsuo, Peters, & Reilly, 1975), allowing the positive corneal potentials to be conducted to the extra-ocular regions. If the insulator hypothesis for blink potentials were valid, the eyelids would block transmission of the positively charged corneal potential to the scalp, and the blink potentials at all EOG electrode sites should therefore be *negative*. On the other hand, if the conduction hypothesis were true, the eyelids would conduct the positive flow from the cornea to the extraocular skin, thus generating *positive* blink potentials at all EOG electrode sites.

However, although our EOG measurements are generally in support of the conduction hypothesis, detailed measures at various EOG electrode sites around the eyes indicate a different scenario. The blink potentials are strongly *positive* for the forehead electrode derivations, but weakly *negative* for the infraorbital derivations, and close to *zero* for the canthi derivations. We conclude that neither the volume conduction nor the insulator hypothesis can fully explain these results, although the sign of the larger forehead signal is consistent with the conduction hypothesis related to conduction through the upper eyelid.

One possible explanation for these results is that the assumption that the eyes are stationary during rapid blinks is incorrect. In principle, if the eyes moved upward to a sufficient extent during the blinks, they could invert the infraorbital signal while more than doubling the forehead signal. According to the calibration data of Fig. 7, such rapid-blink signals would have required an upward eye movement of about 10° (corresponding to a differential signal between the forehead and cheek electrodes of about 160 *μ*V). In the data from Fig. 2, however, the blink signals are as much as 500 *μ*V, implying eye rotations of up to ~ 30° during free blinks.

There are three lines of reasoning against this interpretation, however.

The participant in the blink control study in Fig. 2 was specifically instructed to maintain fixation while performing the rapid blink sequence, and reported no visible movement of the fixation target. Since the eyelids are open to the central target region for most of the lid movement, until just before it closes, it should have been easy for the participant to see any corresponding retinal motion produced by the large eye movements required for this explanation.

Riggs, Kelly, Manning, and Moore (1987, p.334) found that the eyes move very little during blinks when they are close to the primary straight-ahead position: “In normal conditions of viewing there is no evidence of conjugate saccades, or of  any large, upward rotation of the eyes (Bell’s phenomenon) that was once believed to take place during a blink.” (When converged, the eyes tend to move back toward the primary position during blinks, but this is a horizontal rather than a vertical movement, and is thus inconsistent with all our recorded blink signals in the vertically aligned electrodes.) In detail, the Riggs et al. results show that the minimum blink motion for a fixation position slightly nasalward and upward of primary position, but the motion in primary position itself was only about 1°, so it would be drastically insufficient to account for our results with the eyes in primary position.

According to the difference in the time courses of the eye rotation signals and the more rapid blink signals reported by Riggs et al. (1987), the blink responses should appear as a complex difference in the waveforms recorded above and below the eyes during blinks, but no such differences are apparent in Fig. 2.

Given this analysis, the only plausible explanation for the variations in sign of the eye blink responses is that there are *differential insulation/conduction relationships* according to the composition of the eyelids. If the skin operates as an electrical insulator, it could account for the reduction in the cheek signal with closure of the lower lid. If, on the other hand, the levator muscle underlying the upper lid acted as an electrical conductor, it could account for the large increase in the forehead signal, sufficient to overcome any insulator effects. We conclude that the muscle acts as an electrical conductor and the skin acts as an electrical insulator to produce the variety of blink responses that we see from the facial electrode derivations.

## Conclusions

The present eye-tracking capability using EOG signals from a high-density electrode net verifies that EOG can be used for simultaneous measurements of brain responses and eye movements. This capability will be particularly useful for measuring fixation- or saccade-related EEG potentials in order to examine the neural mechanisms involved in the control of eye movements, without the difficulties of synchronization between separate EEG and video-based eye-tracking devices. Further studies aiming to determine fixation- or saccade-related potentials under free-viewing conditions could be based on the present results, further validating the accuracy of calibrated EOG signals for 2-D eye tracking.
